# Targeting Immune-Mediated Dormancy: A Promising Treatment of Cancer

**DOI:** 10.3389/fonc.2019.00498

**Published:** 2019-06-26

**Authors:** Hao-fan Wang, Sha-sha Wang, Mei-chang Huang, Xin-hua Liang, Ya-Jie Tang, Ya-ling Tang

**Affiliations:** ^1^State Key Laboratory of Oral Diseases and National Clinical Research Center for Oral Diseases and Department of Oral and Maxillofacial Surgery, West China Hospital of Stomatology, Sichuan University, Chengdu, China; ^2^State Key Laboratory of Microbial Technology, Shandong University, Qingdao, China; ^3^Hubei Key Laboratory of Industrial Microbiology, Hubei Provincial Cooperative Innovation Center of Industrial Fermentation, Key Laboratory of Fermentation Engineering (Ministry of Education), Hubei University of Technology, Wuhan, China

**Keywords:** cancer dormancy, immune-mediated dormancy, metastases, disseminated tumor cells (DTCs), immunotherapies

## Abstract

Immune-mediated dormancy is when the immune system keeps proliferating tumor cells unchanged, mostly via cytotoxic activity of immune cells. Cancer dormancy, especially immune-mediated dormancy, may be the explanation for tumor refractory and may be responsible for resistance to conventional chemo- and radiotherapies. Here, we will describe different scenarios as to how the immune cells and cytokines involved in cancer progression are connected with the initiation of dormancy and cancer treatment. Two distinct treatment methods, such as maintaining metastatic tumor cells dormant and awakening them, are also discussed. A better understanding of immune-mediated dormancy will help to design novel and effective immunotherapies and will likely increase the efficiency of tumor treatment inhibiting metastasis.

## Introduction

In cancer progression, there is a phase for tumor cells to impair non-primary organs due to the formation of overt metastases, which is the major cause of cancer-related deaths (1). Cancer recurrence and metastases have been confirmed to be associated with cancer dormancy (2), which refers to the clinically asymptomatic period after treatment of the primary tumor. Cancer dormancy may be one of the explanations for tumor refractory and may be responsible for the phenomenon that malignancies cannot be cured by the initial treatment of primary tumor (3). Thus, a comprehensive understanding of cancer dormancy will be urgently needed to help the development of cancer immunotherapies, which might represent a promising approach against cancer metastases.

As early as 1934 and 1954, Willis (4) first proposed the dormancy of tumors and then Hadfield (5) defined cancer dormancy as a state of temporary mitotic arrest. Decades later, cancer dormancy has been further explored and has been divided into three types: cellular dormancy, angiogenic dormancy, and immune-mediated dormancy (2). Cellular dormancy is generally considered to precede the angiogenic dormancy or the immune-mediated dormancy phase, and these three models are not mutually exclusive (2). Quiescence from a G0-G1 arrest (6) could be a more plausible mechanism to define cancer dormancy. Quiescence is reversible, a property similar to that of dormant tumor cells (2, 7). Dormant disseminated tumor cells (DTCs) have tumorigenic and metastatic potency, which could be the origin of future metastases (8). Dormant DTCs in bone marrow of breast cancer patients were associated with a poor prognosis and increased recurrent risk (9, 10). The treatment aiming at dormant DTCs has been raised as a means of metastasis prevention.

One of the essential properties of dormant DTCs is their resistance to chemotherapy (2, 11). There are two main hypotheses to explain this resistance. The first assumption is that dormant DTCs could be resistant to cytotoxic therapies targeting rapidly dividing cells, since dormant DTCs are not dividing (2). The second is that dormant DTCs are further evolved than the primary tumor cells and have accumulated mutations to be more refractory to treatment (11). P38 signaling in dormant cells could activate the endoplasmic reticulum (ER) chaperone, BiP, and RNA-dependent protein kinase–like ER kinase (PERK), which protect dormant tumor cells from chemotherapy (12). Presently, distant strategies targeting dormant DTCs are killing them with the potential risk of waking them up or keeping them “asleep” forever. However, cancer dormancy clinically appears as a persistent disease without symptoms or signs, unless the balance is disturbed, just like the complete control of a chronic disease (13). Keeping dormant DTCs “asleep” continues to appear to be a more promising strategy (7).

**Figure 1:**
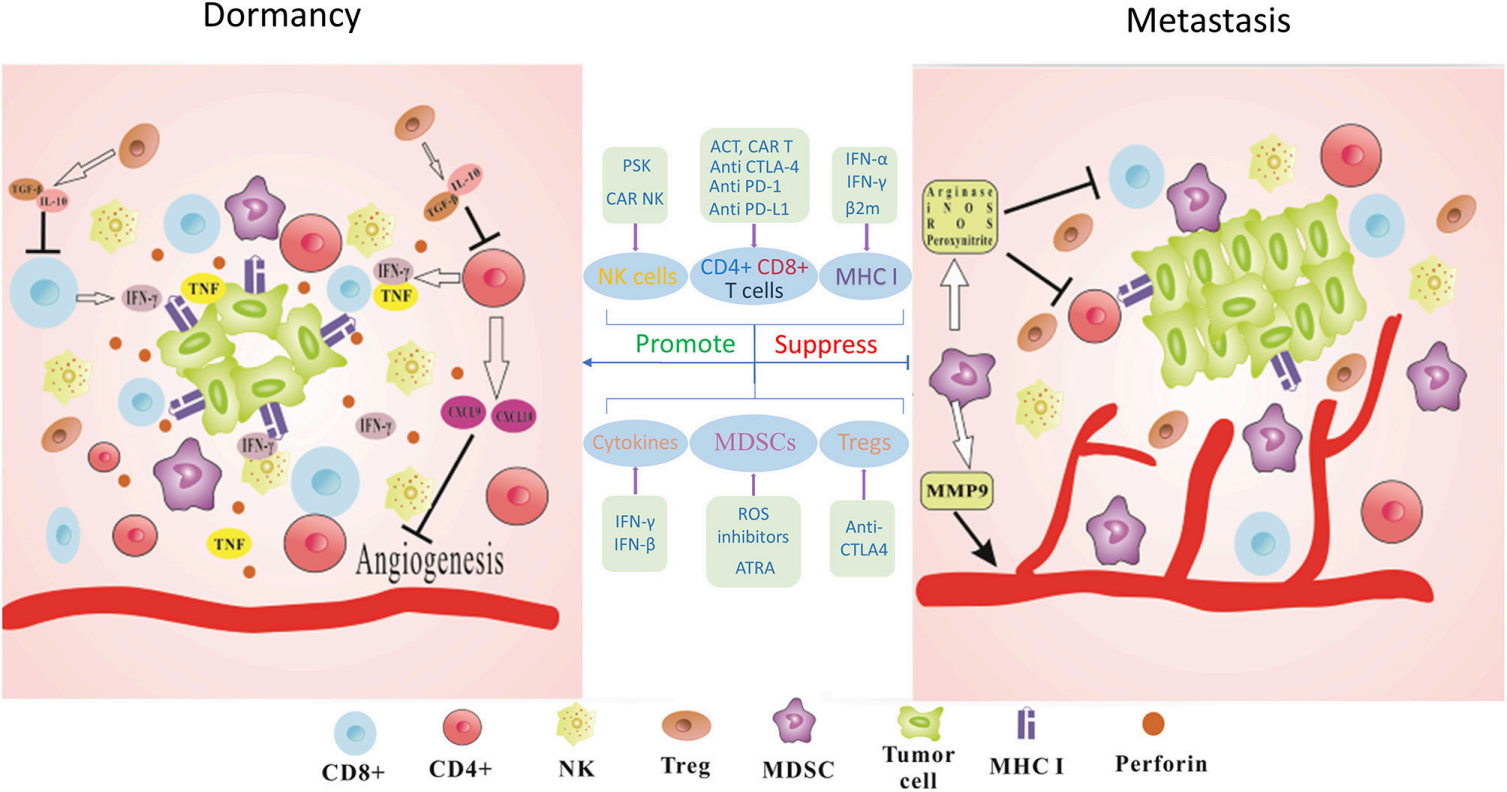
Immune-mediated dormancy. The maintenance of functional dormancy in tumor cells requires the combined action of many immune factors. IFN-γ and TNF derived from T cells could directly regulate cell cycle progression of tumor cells and establish the dormant state. CD4+ T cells released antiangiogenic chemokines CXCL9 and CXCL10 leading to reduced angiogenesis and antitumor effects. Perforin-mediated cytotoxicity of NK cells also keeps tumor cells in dormant state and prevents the outgrowth of metastasis. MHC I surface expression on tumor cells could be also involved in this process. Furthermore, immunosuppressive cytokines such as TGF-β or IL-10 produced by Tregs could lead to decreased activity of T cells. MDSCs act as potent suppressors of T cells via the production of Arginase, iNOS, ROS, and Peroxynitrite. MDSCs could secret MMP9 to promote tumor angiogenesis and facilitate tumor development. Thus, blocking the immunosuppressive checkpoints PD-1, PD-L1, and CTLA-4 to activate CD8+ and CD4+ T cells, applying PSK or CAR-NK cells to augment the infiltration of NK cells, using IFN-γ treatment to upregulate or recovery MHC I surface expression, suppressing ROS and CTLA-4 to deplete MDSCs and Tregs, interfering with cytokine production to modify tumor microenvironment could be promising immunotherapeutic strategies for cancer metastasis.

Since the dual role of the immune system in cancer progression has been well-established, immunoediting was proposed to describe host-protective and tumor-promoting roles of immune system (14). Immunoediting could be explained by three mechanisms: elimination, where developing cancers are eradicated by the interaction of innate and adaptive immunity long before they become clinically apparent; equilibrium, where tumor cells maintain in a state of functional dormancy, due to the balance of anti-tumor and tumor promoting factors; escape, where the outgrowth of tumor cells is no longer attenuated by immunity, resulting in the induction of immunosuppressive tumor microenvironment and clinically apparent diseases (15). Immune-mediated dormancy could be regarded as the state of equilibrium, because both result from the balance of pro-tumor and anti-tumor functions of the immune system. Two recipients without primary melanoma received a kidney from the same donor who had previously had primary melanoma but was thought to have no residual tumor and to be apparently tumor free 15 years after surgical removal; however, both of these recipients were diagnosed with secondary melanoma in the donated kidney after immunosuppressive therapy (16), implying that the immune system could keep tumor cells dormant in non-primary organs. In a mouse model of 3′-methylcholanthrene (MCA) induced sarcomas, the equilibrium or dormant state of tumor, which was characterized by a combination of decreased proliferation and increased apoptosis of tumor cells, was maintained by adaptive immune components, including CD4/CD8 T cells, interferon (IFN)-γ and interleukin (IL)-12 (17). In addition to those mentioned above, some other immune factors, including myeloid-derived suppressor cells (MDSCs), Treg cells, natural killer (NK) cells, MHC class I surface expression and several cytokines also have played important roles in controlling dormant DTCs. Besides, some non-immune mechanisms may be used by the immune system to promote dormancy (18) (Figure 1). Thus, we will focus on the cells and cytokines involved in immune-mediated dormancy and propose several immunotherapies targeting cancer dormancy. A better understanding of immune-mediated dormancy would help to design novel and effective cancer immunotherapies.

## Effector T Cells

Effector T cells, especially CD8+ and CD4+ T cells, get the most attention in cancer dormancy. CD8+ T cells are the preferred lymphocytes to suppress tumors due to their incomparable ability of recognizing intracellular antigens expressed by all tumor cell types. CD4+ T cells could destroy tumor cells through cytolytic mechanisms or modulate tumor microenvironment to exert their anti-cancer role. Besides, CD4+ T cells are known for their capacity to help CD8+ T cells to overcome negative regulation and enhance their antitumor responses (19). In a spontaneous mouse model of melanoma, while tumor cell dissemination was an early event in progression of primary tumor, the development of overt metastases in the lung was postponed due to dormant DTCs, and metastases in lung were more rapid in CD8-depleted mice, which indicated that reduced proliferation or dormant maintenance of DTCs needed the involvement of cytostatic CD8+ T cells (20). Consistent with this finding, in a mouse model of tumor dormancy, the depletion of the CD8+ T cells was significantly associated with decreased duration of dormancy and shortened mean time for B cell lymphoma recurrence on the spleen, showing that CD8+ T cells contributed to the induction and maintenance of the state of dormancy via production of IFN-γ (21). In both *vitro* and *vivo*, IFN-γ produced by cytotoxic T lymphocytes (CTLs) could induce G0/G1 arrest and the dormancy of tumor-repopulating cells (TRCs), which refer to a subpopulation of cancer cells with the capacity of self-renewing and highly tumorigenic in several types of murine and human tumors, such as melanoma and liver cancer. IFN-γ mediates TRCs dormancy via an indolamine 2,3-dioxygenase 1 (IDO1)-kynurenine (Kyn)-aryl hydrocarbon receptor (AhR)-p27 pathway. In particular, p27 binding could inactivate JAK-STAT1 pathway, a signaling that may induce apoptosis and disrupt the dormancy program. Besides, the induction of TRCs dormancy was only found at high concentrations of IFN-γ (>50 ng ml^−1^), while low dose (20 ng ml^−1^) could maintain dormancy, suggesting that different mechanisms appeared to be involved in dormancy induction and maintenance (22). In a mouse model of acute myeloid leukemia, expression of B7-H1 (also known as PD-L1, the ligand for PD-1) or B7-1 (also regarded as CD80, the ligand for CTLA-4) could contribute to the prolonged persistence of tumor cells via inhibiting CD8+ T cells-mediated killing (23), which pointed out another possible mechanism in which CD8+ T cells are involved in cancer dormancy.

In breast cancer patients (24) and a mouse lymphoma model (25), dormant DTCs have been observed to persist in the bone marrow along with a rise in CD4+ and CD8+ T cells, indicating that in addition to CD8+ T cells, CD4+ T cells also played a role in cancer dormancy. CD4+ T cells (Th1 cells) combined with IFN-γ signaling and tumor necrosis factor p55 receptor (TNFR1) signaling could arrest tumor growth and establish a state of tumor dormancy in an analyzing T antigen (Tag)-induced pancreatic cancer mouse model. Furthermore, the release of antiangiogenic chemokines, CXCL9 and CXCL10, and aberrant expression of αvβ3 integrin caused by CD4+ T cells, which resulted in decreased formation of tumor vessel, also contributed to the induction of cancer dormancy, suggesting that the immune system could be involved in cancer dormancy, not only through immune mechanisms but also through non-immune mechanisms such as regulating tumor angiogenesis (26). Furthermore, a higher ratio of CD8+/CD4+ T cells was observed in mice with dormant sarcomas compared with mice with progressing sarcomas, suggesting that increased CD8+/CD4+ T cells ratio may contribute to maintain an equilibrium state and cancer dormancy (27). Using a fibrosarcoma mouse model, which was characterized by maintaining the capacity of metastases in a state of permanent immuno-mediated dormancy without additional antitumor therapies, spontaneous pulmonary metastases were developed in 100% of mice depleted of CD8+ T cells, and in 23% of those depleted of CD4+ T cells (28). This study may draw a conclusion that, in terms of restraining spontaneous metastases in permanent dormancy, CD8+ T cells seem to be more important than CD4+ T cells. However, Braumüller et al. (29) proposed that the combination of IFN-γ and TNF, derived from Th1 cells, drove cancer cells into senescence by arresting cells in G1/G0 which was irreversible, unlike quiescence. And this cytokine-mediated senescence required the stabilization of the p16INK4a–Rb pathway and the combined action of STAT1 and TNFR1 signaling. Much on the relationship between quiescence and senescence induced by CD4+ T cells should be done.

As noted above, CD8+ and CD4+ T cells, albeit the effects are different, involve in cancer dormancy and metastases prevention via both immune and non-immune mechanisms. Thus, activation of these cells could be an effective anti-tumor immunotherapy, which has been proven by clinical observation showing that high levels of CD8+ and CD4+ T cells are associated with improved survival in breast (30), head and neck (31), colon (32), and lung cancer (33). One of the immunotherapies aiming at activating these cells is to block the immunosuppressive checkpoints expressed on cancer cells or on T cells, such as PD-1, PD-L1, and CTLA-4 (18). Specifically, PD-L1 expressed by tumor cells interacted with PD-1 on T cells leading to increased apoptosis of T cells and T cell exhaustion. And expression of CTLA-4, which is highly homologous to CD28, could be induced by T cell activation. CTLA-4 could bind to B7 molecules on antigen-presenting cells (APCs) with much higher affinity than CD28, resulting in the accumulation of CTLA-4 in the T cell at the T cell-APC interface and the eventual abrogation of activated T cell response (34). In fact, nivolumab and pembrolizumab, PD-1 inhibitors, have received licensure for the treatment of patients with recurrent and/or metastatic HNSCC from the U.S. Food and Drug Administration (FDA) (35). In a randomized phase 3 trial, 361 patients with recurrent HNSCC were divided into two groups: the nivolumab group and the standard-therapy group (methotrexate, docetaxel, or cetuximab). The median overall survivals of the two groups were 7.5 and 5.1 months, respectively, and the response rates were 13.3 and 5.8%, respectively (36). The FDA granted approval for nivolumab in combination with ipilimumab, a CTLA-4 inhibitor, for the treatment of patients with BRAF V600 wild-type unresectable or metastatic melanoma in 2014 (37). In a clinical study, the confirmed objective response rate of BRAF V600 wild-type metastatic melanoma patients, who received the combination therapy of nivolumab and ipilimumab, was 61.1% (44/72) compared with 10.8% (4/37) in the ipilimumab monotherapy group. Besides, the complete response rate was 22.2% (16/72) of patients in the combination group, whereas in the ipilimumab group, none were reported to receive complete responses (38).

Another promising cancer immunotherapy is adoptive cell transfer (ACT), which refers to the infusion of lymphocytes into a tumor host after their stimulation and expansion *in vitro* to exert an anti-tumor effect (39). Adoptive cell therapy using autologous TILs has been regarded as the most effective treatment for patients with metastatic melanoma to receive complete lasting regression (40). In a phase 2 study, which enrolled 21 metastatic melanoma patients, 20 evaluable patients received TIL therapy. Seven of them (35%) were found to have received objective tumor regression, where six patients achieved partial response and one patient achieved complete response at 21 months post therapy (41). Chimeric antigen receptors (CAR) are composed of a tumor associated antigen binding region [usually derived from the single-chain variable fragment (scFv) segment of the monoclonal antibody], an extracellular hinged region, a transmembrane region, and an intracellular region. CAR T cells have an incomparable antitumor advantage, in as much as the independence of CAR recognition from MHC restriction. FDA has approved autologous T cells engineered to express a CAR targeting CD19 for the treatment of refractory pre-B cell acute lymphoblastic leukemia and diffuse large B cell lymphoma (42). In a phase 1 trial involving 53 patients with relapsed B-cell acute lymphoblastic leukemia received CD19-specific CAR T cells, and 44 patients (83%) had a complete remission (43). However, CAR-based therapy in solid tumors has made limited progress (42).

## Natural Killer(NK) Cells

NK cells as pivotal component of innate immunity could induce the death of tumor cells mainly via cytotoxicity and the production of cytokines. Whereas, Koebel et al. (17) proposed that the maintenance of equilibrium was solely associated with adaptive immunity, Nair et al. (44) found that latency competent cancer (LCC) cells could enter a quiescent state and remain latent in primary and metastatic organs for extended periods by evading innate immune surveillance, especially NK cell-mediated clearance. Quiescent LCC cells expressed dickkopf-related protein 1 (DKK1), a WNT inhibitor, leading to board downregulation of NK cell activating ligands UL16-binding proteins (ULBP) and decreased cytotoxicity of NK cells (45). Compared with the percentages in mice with progressing sarcomas, mice with dormant sarcomas had significantly higher percentages of NK cells (27). Brodbeck et al. (46) used a mouse model of colon cancer, finding the vital role of NK cells in both the growth of a primary tumor and formation of distant metastases. Then they utilized a computer modeling for further analysis, suggesting that perforin-mediated cytotoxicity of NK cells could force DTCs to maintain in dormant state for at least 30 days through restraining their proliferation. Saudemont et al. (47) proposed that CXCL10 could not only induce an efficient immune response, but could also clear DTCs resistant to CTL-mediated killing in order to cure acute myeloid leukemia, which was completely dependent on NK cells, and partially dependent on CD4+ and CD8+ T cells. This may be due to the expression of PD-L1 on NK cells, which could stimulate the proliferation and the production of IFN-γ and TNF-α by CD4+ and CD8+ T cells. However, they also suggested that this effect of PD-L1+ NK cells was not caused by binding to PD-1.

NK cells play a significant role in cancer dormancy, where their activator function triggering T lymphocytes response seems to be more important than the direct cytotoxic capacity. NK cells could not only maintain dormant state, but also destroy dormant DTCs, thus the activation of NK cells could be another potential immunotherapy targeting cancer dormancy (18). The treatment of protein-bound polysaccharide K (PSK), although it had no cytotoxic effect on murine fibrosarcoma tumor cells, could markedly augment the infiltration of NK cells leading to all injected mice becoming metastasis-free and demonstrating a favorable therapeutic effect of eradication of metastases (48). Cancer therapies targeting activating NKG2D, a major activating receptor for NK cells, has been shown to improve NK cell responses leading to the suppression of tumor growth and the reduced formation of metastases in various tumor types, such as melanoma, osteosarcoma and hepatocellular carcinoma (49). CAR-NK cells could be an alternative approach to receive more potent antitumor activity and less side effects (50). A study used CAR-NK cells to treat three patients with metastatic colorectal cancer. Two of them demonstrated reduced ascites generation and a markedly decreased number of tumor cells in ascites samples, and the third patient with hepatic metastases was observed to have rapid tumor regression in the liver (51).

## Regulatory T Cells (Tregs)

Tregs in tumor microenvironment have been shown to be associated with immune suppression and tumor progression in several types of human cancer, such as colorectal (52), head and neck cancer (53), ovarian (54) and gastric cancer (55). Tregs were also found to play a role in inhibiting the proliferation of tumor-specific effector T cells (especially CD8+ and CD4+ T cells) and suppressing the secretion of IFN-γ and IL-2 by effector T cells via expressing intracellular CTLA-4, glucocorticoid—induced tumor necrosis factor receptor (GITR) and FOXP3(54). In addition, the production of immunosuppressive cytokines, such as transforming growth factor (TGF)-β or IL-10 by Tregs, and the direct interaction between Tregs and effector T cells, could damage the activity of effector T cells (56). However, different dormant tumors were found to have different infiltrations of Tregs. Compared with progressing sarcomas, the level of Tregs was significantly lower in dormant sarcomas (27). In contrast, the organ harboring dormant tumor cells had higher number of Tregs than the organ with actively growing tumor cells in the mouse B cell lymphoma model (BCL1), whereas, Tregs from both tumor microenvironments had similar capacities in suppressing the proliferation of CD4+ and CD8+ T cells suggesting the function of Tregs was not impaired by the induction of dormancy (57). Due to the role of Tregs in suppressing the activity of CD8+ and CD4+ T cells, the maintenance of an equilibrium state was influenced by higher CD8+/Treg cell ratio, which was also associated with improved survival of sarcomas mice (27).

Although the infiltration and the function of Tregs in tumor microenvironment are still obscure, cancer treatments targeting Tregs have made some progress. Anti-CTLA4 therapy could decrease the population and activity of Tregs, contributing partially to antitumor efficacy (58), which has been supported in murine colon adenocarcinoma tumor models and bladder cancer patients (59, 60). In a randomized, double-blind, phase 3 study, 273 patients with unresectable metastatic melanoma were randomly distributed to receive ipilimumab alone or glycoprotein 100 (gp100) peptide vaccine alone. The median overall survivals of ipilimumab alone group and gp100-alone group were 10.1 and 6.4 months, respectively. The rate of overall survival in ipilimumab-alone group was significantly higher than that in gp100-alone group (45.6, and 25.3% at 12 months, respectively) (61).

## Myeloid-Derived Suppressor Cells (MDSCs)

MDSCs have been shown to play a prominent role in the establishment of pre-metastatic niches which contribute to the re-activation of dormant DTCs and support subsequent metastatic outgrowth (62–64). MDSCs represent a population of special cells of the immune system, which consist of immature macrophages, immature granulocytes and immature dendritic cells. Their remarkable ability is to act as potent suppressors of T cell responses via direct cell–cell contact or the production of Arginase, iNOS, ROS, and Peroxynitrite. Furthermore, another possible capacity of MDSCs is to promote Tregs expansion through the production of cytokines, such as IFNγ and IL-10 (65). MDSCs could directly contact with NK cells to suppress IL-2–mediated NK cell cytotoxicity (66). These are the indirect evidences to show the association of MDSCs with cancer dormancy, and at present, there are only a few reports to show the direct relation between them. MDSCs could oppose the tumor suppressor gene (Pten) via the release of interleukin-1 receptor antagonist (IL-1RA) with the capacity of interfering with IL-1α signaling and impairing oncogene-induced senescence, thereby leading to senescence evasion and tumor development (67). In addition to their immunosuppressive ability, MDSCs could contribute to tumor angiogenesis primarily via the secretion of MMP9, which increased the bioavailability of VEGF, thereby destroying angiogenic dormancy and facilitating tumor metastasis (68, 69).

The immunotherapies targeting MDSCs could be divided into three major strategies: inactivating MDSCs, depleting MDSCs, or converting MDSCs into mature myeloid cells and APCs without suppressive abilities (65, 70). There are many ways to achieve the elimination and inactivation of MDSCs, such as ROS inhibitors and gemcitabine (65). In addition, all-trans-retinoic acid (ATRA) has been proven to promote MDSCs to differentiate into mature myeloid cells (71, 72). ATRA could cause the induction of dormant DTCs in HNSCC via p38 MAPK-dependent pathway (73). In addition, the combination of ATRA and natural killer T (NKT) cells activation was shown to convert immunosuppressive MDSCs into immunogenic APCs via the secretion of IFNγ by NKT cells and the upregulation of ATRA-mediated glutathione synthase (GSS) (74).

## MHC Class I Expression

MHC I molecules, which are famous for their ability to present tumor antigens to T lymphocytes and regulate NK cell function, have been proved to be involved in immune escape of cancer cells (75) and act as tumor suppressors (76). Using a tumor dormancy-derived cell line, MHC I expression was found to be upregulated on the surface of dormant tumor cells, compared with the parental cell lines (25). Consistent with this finding, in a fibrosarcoma mouse model, the interaction between MHC I molecules and immune cells, especially CD8+ T cells, could promote metastatic cells into dormant state, due to the immunoregulation and tumor suppression of MHC I (28). However, Pantel and his colleagues draw an opposing conclusion about the expression of MHC I on DTCs. They measured MHC class I expression on DTCs from bone marrow aspirates of patients with different adenocarcinomas (including breast, stomach, and colon) and concluded that quiescent micrometastasis cells in the bone marrow had reduced MHC class I expression, which may be in favor of their survival and subsequent outgrowth (77). In terms of these different observations, there was a possible explanation that a dynamic and interchangeable equilibrium state between MHC-I–positive and MHC-I–negative cells may exist to accommodate complex signals changes in tumor microenvironment (28). Although the expression of MHC I on DTCs is controversial, all of these authors regarded upregulating or recovering MHC I surface expression as an effective antitumor treatment.

The alterations in MHC I expression in cancers could be divided into two types: reversible defects (regulatory or “soft”) and irreversible defects (structural or “hard”). “Soft” MHC I defects could be recovered after the treatment of cytokines, such as IFN-α and -γ, or immunotherapies to increase the production of these cytokines, whereas “hard” MHC I defects could not be recovered. Because irreversible structural abnormalities of MHC I were caused by loss of heterozygosity (LOH) of MHC-associated genes, correction of this defect can only be achieved by the transference of a wild type MHC I heavy chain or by β2-microglobulin (β2m) genes (78). To the best of my knowledge, there are no clinical reports on targeting MHC I in immune-mediated dormancy.

## Cytokines

Cytokines, including IL, IFN and TGF, are proteins, peptides or glycoproteins secreted by immune cells, which could aid cell to cell communication in immune responses. Several cytokines have also been shown to be involved in immune-mediated dormancy.

IFN-γ not only has antitumor function mediated by immune cells, but also has tumor promotion ability determined by chronic inflammation (79). As mentioned above, IFN-γ mainly produced by effector T cells and NK cells could be involved in cancer dormancy via STAT1 signaling (79). IFN-γ induced melanoma cells into G0/G1 growth arrest and the state of dormancy, which was associated with decreased cyclin A, cyclin E and certain cyclin-dependent kinases (CDK2 and CDK4). STAT1, which could interact directly with the cyclin D1/CDK4 complex (80), was an essential mediator of this process (81). Besides, IFN-γ could upregulate the expression of MHC I with the help of STAT1 signaling (82), pointing out another possible mechanism of IFN-γ to mediate cancer dormancy.

Cell-cycle analysis for mouse melanomas indicated that IFN-β treatment could induce 72.4% tumor cells into the state of G0/G1 arrest, compared with 20.5% quiescent tumor cells from the control group, suggesting that IFN-β could induce melanoma cells into dormancy. Further research showed that IFN-β mobilized the IDO1/Kyn/AhR/p27-dependent pathway to mediate tumor cells dormancy. Furthermore, a combination of IDO1 or AhR inhibitors to block this pathway and IFN-β treatment significantly decreased colony size and colony number of tumor cells, due to dormant tumor cells that were abrogated, which provided a novel and ideal treatment against cancer dormancy (83).

In an MCA-induced mouse sarcoma model, both IL-23 and IL-12 were found to contribute to maintain cancer cells in the state of dormancy, whereas their roles were completely opposite. IL-23, with the capacity of promoting cancer persistence, played a critical role in opposing the effects of IL-12 with the ability of preventing cancer outgrowth. A combined treatment of anti-IL23p19 and anti-CD40, which could stimulate APC to produce IL-12p70, may help to eliminate persistent cancer cells in equilibrium lesions (84).

In a HNSCC model, TGFβ2 signaling in the bone marrow was found to decrease ERK/p38 activity ratio, which predicted whether tumor cells would enter a state of dormancy (85), thereby inducing the expression of DEC2, which was related with dormancy and quiescence. DEC2 overexpression in tumor cells resulted in the expression of p27, which eventually led to dormancy of DTCs (86). However, in a mouse breast cancer model, increased level of TGFβ1 was associated with extensive deposition of type I collagen (Col-I) and pulmonary fibrosis, and then with activation of β1-integrin signaling and induction of the dormant-to-proliferative switch of DTCs (87), suggesting that TGFβ1 may play an opposite role in cancer dormancy, compared with TGFβ2.

## Epilogue

All of the evidences and experiment researches mentioned above indicate that the immune system may play a central role in cancer dormancy. These clinical trials mentioned have shown that immunotherapies have achieved partial success in patients with overt metastatic diseases or recurrent diseases. Especially, immunotherapies aiming at activating CD8+ and CD4+ T cells or reversing their immunosuppression may be effective to restrain tumor metastasis. However, when it comes to transplanted patients or those with autoimmune disease, the immunosuppressive treatments must be prudent to administer for avoiding awakening dormant metastases (18).

In addition, the combined therapies are also one of the most promising treatments. For example, chemotherapy could increase the susceptibility of tumor cells to cytotoxic T lymphocytes, and the administration of chemotherapy and immunotherapy could help to improve the immunosuppressive trait of cancers (88). A mouse model suggested that the combination of chemotherapy or immunotherapy and adoptive T cell therapy could lead to the eradication of established tumors via destroying cancer stroma (89). Lower-dose antiangiogenic treatment (anti-VEGF therapy) could normalize breast tumor vasculature and change the tumor microenvironment from being immunosuppressive to immunosupportive, thereby improving the efficacy of immunotherapies (90). Therefore, in addition to the development of new drugs or novel treatments, the optimal doses, sequences and timing of combined therapies should also be the focus of future researches. Rational clinical researches to measure dormancy therapies are extremely urgent. Treatments mentioned above mainly aim at cancer patients with overt metastatic diseases or recurrent diseases; however, little progress seems to have been made in effective therapies targeting individuals with suspected latent disseminated diseases. Another issue that needs to be considered in the development of treatments targeting dormancy is over-treatment, which may lead to cancer recurrence. A greater understanding of immune-mediated dormancy is fundamental for solving these difficulties.

## Author Contributions

All authors listed have made a substantial, direct and intellectual contribution to the work, and approved it for publication.

### Conflict of Interest Statement

The authors declare that the research was conducted in the absence of any commercial or financial relationships that could be construed as a potential conflict of interest.
